# Editorial: Advances in the understanding and treatment of cutaneous lymphoma

**DOI:** 10.3389/fonc.2023.1190269

**Published:** 2023-03-28

**Authors:** Andrea Bernardelli, Lorenzo Falchi, Jianjun Qiao, Carlo Visco

**Affiliations:** ^1^ Department of Engineering for Innovation Medicine, Section of Hematology, University of Verona, Verona, Italy; ^2^ Lymphoma Service, Department of Medicine, Memorial Sloan-Kettering Cancer Center, New York, NY, United States; ^3^ Department of Dermatology, The First Affiliated Hospital, Zhejiang University School of Medicine, Hangzhou, China

**Keywords:** skin cancer, non Hodgkin’s lymphoma, primary cutaneous lymphoma, cutaneous T-cell lymphomas, cutaneous B-cell lymphomas, mogamulizumab, PCL, JAK/STAT

Primary cutaneous lymphomas (PCL) are a heterogeneous group of non-Hodgkin’s lymphomas characterized by the clonal proliferation of skin-homing malignant T- or B-cells. Among them, cutaneous T-cell lymphomas (CTCL) are the most frequently encountered in clinical practice, with mycosis fungoides/Sezary syndrome (MF/SS) being the most prevalent subtype. Primary cutaneous B-cell lymphomas (PCBCL) account for approximately 25% of all PCL and are classified into three major subgroups: primary cutaneous marginal zone lymphoma (PCMZL), primary cutaneous follicle-center cell lymphoma (PCFCL), and diffuse large B-cell lymphoma, leg type (PCDLBCL, LT) (1).

Progress has been made in understanding the molecular pathogenesis of both CTCL and PCBCL, leading to the development of new therapeutic strategies for disease control. The collection of papers in this Research Topic provides a description of some of the current advances in the understanding and treatment of cutaneous lymphomas.

In a mini-review, Vitiello et al report on the clinical heterogeneity of PCBCLs, and focus on treatment decisions. It is discussed how accurate histological and immunophenotypic classification of the lymphoma subtype allows for individual risk analysis. Both PCMZL and PCFCL are classified as indolent lymphomas with 5-year disease-specific survival rates exceeding 95%. Conversely, PCDLBCL, LT is an aggressive, difficult-to-treat lymphoma with specific clinical-pathological characteristics, and represent a significant medical unmet need.

Two reviews focus on key pathogenetic aspects of CTCL. Bakr and Whittaker review the key pathways disrupted in CTCL, and discuss the potential therapeutic implications of these findings. SS in particular, is characterized by a high heterogeneity, and recent studies have shown the presence of subpopulations with different gene expressions and different sensitivity to treatment with histone deacetylase inhibitors (HADCi).

Zhang and Zhang describe the central role of epigenetics in CTCL. Despite ongoing research, the pathogenesis of MF/SS remains a complex process that is not yet fully understood. Due to the lack of animal models of CTCL, single-cell sequencing has been applied to explore the pathogenetic pathways that characterize CTCL. Epigenetic changes play a role in malignant transformation and HDACi resistance in CTCL, prompting the need of deciphering novel HDACi targets in the disease. It is known that *SOCS1* is one of the genes frequently mutated in the early stages of MF, which plays a negative regulatory role in the *JAK/STAT* pathways, that may be actively targeted by drugs.

In their review, Fay et al underline the crucial role of the native immune system in lymphoma progression. Understanding the immune effects of therapies may facilitate the development of new agents that leverage the immune system for the treatment of CTCL. The role of monoclonal antibodies, PD-L1 inhibitors, immunomodulators, brentuximab vedotin, and mogamulizumab are discussed. In particular, mogamulizumab induces antibody-dependent cellular cytotoxicity and complement-dependent cytotoxicity, leading to the destruction of CCR4-expressing cancer cells. Combination therapies, such as mogamulizumab plus radiation therapy (NCT04128072 or NCT04256018) or brentuximab vedotin plus pembrolizumab (NCT05313243), are also covered. Furthermore, mutations affecting the NFkB pathway, DNA damage response, chromatin modification, and *JAK/STAT* pathways are described as potential target for future therapies.

Finally, two original papers are included in this series. Luo et al. report on the creation of an *in vivo* autochthonous murine model with *SOCS1* deletion in CD4 T-lymphocytes that infiltrate the skin. Immunohistochemical analysis of skin biopsies from these mice showed that *SOCS1* deletion promotes an inflammatory state and leads to *STAT3* activation, and subsequent creation of a lymphoma phenotype. This work sheds light on the oncogenesis of MF and provides a basis for more advanced genetically engineered mouse models, which can serve as experimental models for development of targeted therapies.

Finally, Peiffer et al, contribute a brief research report using combined single cell RNA and T-cell receptor (TCR) sequencing (scRNA-seq). Comparing the transcriptome of single cells in the blood and skin of a patient with SS, they show different gene expressions in SS cells at the blood and skin level. Using scRNA-seq authors detected a high degree of functional heterogeneity within the malignant T-cell population in SS. The presence of two distinct phenotypes in blood and skin, which are driven by disparate microenvironment-mediated stimuli, could explain the different responses to treatment of the tissue compartments.

Much work remains to be done in understanding the pathogenesis of PCL, and generating new treatment options. For example, the development of biomarkers that can predict patient response or resistance to specific treatments would be instrumental in tailoring treatment strategies. On the other hand, deeper understanding of the pathogenesis of these neoplasms has opened avenues for therapeutic interventions that may offer new hope for better outcomes in patients with diagnosed with PCL.

We express our gratitude to the authors for their valuable articles and contributions to the field, which significantly enhances our understanding and knowledge on the subject matter.

## Author contributions

AB and CV wrote the first draft of the manuscript. All authors contributed to manuscript revision, read, and approved the submitted version
